# Healthcare providers’ awareness and knowledge of recommendations on cardiovascular risk management in people with rheumatic and musculoskeletal diseases: a survey study

**DOI:** 10.1016/j.ero.2026.02.015

**Published:** 2026-03-13

**Authors:** Dina Husum, Julie Katrine Karstensen, Massimo Radin, Savino Sciascia, Alessia Alunno, Fabiola Atzeni, Javier Rodríguez-Carrio, Michael T. Nurmohamed, Sander I. van Leuven, Zoltán Szekanecz, Jette Primdahl, Karen Schreiber, EULAR CardioVascular Involvement in Inflammatory Arthritis (CVIIA) Study Group

**Affiliations:** 1 Danish Center for Expertise in Rheumatology (CeViG), Danish Hospital for Rheumatic Diseases, University Hospital of Southern Denmark, Soenderborg, Denmark; 2 Department of Cardiology, University Hospital of Southern Denmark, Aabenraa, Denmark; 3 Department of Regional Health Research, University of Southern Denmark, Odense, Denmark; 4 University Center of Excellence on Nephrologic, Rheumatologic and Rare Diseases (ERK-Net, ERN-Reconnect and RITA-ERN Member) with Nephrology and Dialysis Unit and Center of Immuno-Rheumatology and Rare Diseases (CMID), Coordinating Center of the Interregional Network for Rare Diseases of Piedmont and Aosta Valley, San Giovanni Bosco Hub Hospital, Department of Clinical and Biological Sciences, University of Turin, Turin, Italy; 5 Department of Clinical Medicine, Public Health, Life and Environmental Sciences (MeSVA), Internal Medicine and Nephrology Division, University of L’Aquila, L’Aquila, Italy; 6 Rheumatology Unit, Department of Internal Medicine and Experimental Medicine, University of Messina, Messina, Italy; 7 Area of Immunology, Department of Functional Biology, Faculty of Medicine, University of Oviedo, Oviedo, Spain; 8 Department of Metabolism, Instituto de Investigación Sanitaria del Principado de Asturias (ISPA), Oviedo, Spain; 9 Reade, Amsterdam Rheumatology and Immunology Center, Amsterdam, The Netherlands; 10 Amsterdam University Medical Center, Department of Rheumatology and Clinical Immunology, Amsterdam, The Netherlands; 11 Department of Rheumatology, Radboud University Medical Center, Nijmegen, The Netherlands; 12 Department of Rheumatology, Faculty of Medicine, University of Debrecen, Debrecen, Hungary; 13 Hospital of Southern Jutland, University Hospital of Southern Denmark, Aabenraa, Denmark; 14 Thrombosis and Haemostasis, Guy’s and St Thomas NHS Foundation Trust, London, UK

## Abstract

**Objectives:**

Patients with rheumatic and musculoskeletal diseases (RMDs) have increased cardiovascular (CV) risk. European Alliance of Associations for Rheumatology (EULAR) issued CV risk management recommendations in 2015/2016 and 2022, but healthcare providers’ (HCPs’) awareness and their implementation in clinical practice are unknown. This study aims to assess HCP awareness of the EULAR CV recommendations and barriers to their implementation.

**Methods:**

A cross-sectional survey was distributed via social media to all HCPs involved in treating patients with RMDs. Data on demographics, awareness of the 2015/2016 and 2022 recommendations, and perceived implementation barriers were collected. The survey was endorsed by the EULAR Study Group on Cardiovascular Involvement in Inflammatory Arthritis.

**Results:**

Of 226 complete responses, 54% were hospital rheumatologists and 36% had 5 to 15 years of experience. Overall, 67% knew both recommendation sets. Knowledge scores were higher among those aware of both recommendations compared with those who knew neither. Most respondents (83%) recognised that platelet inhibitors are not recommended for primary prevention; only 8% identified the role of low systemic lupus erythematosus disease activity. Nearly all (96%) endorsed corticosteroid minimisation. Half viewed rheumatologists as primarily responsible for CV risk management; 16% knew the 1.5 multiplication factor for rheumatoid arthritis risk scoring. None correctly noted that antihypertensives and statins can be used as in the general population. Barriers included time constraints (62%), insufficient knowledge (37%), and lack of local protocols (14%).

**Conclusions:**

Despite moderate awareness of EULAR CV recommendations, substantial knowledge gaps and practical barriers persist, indicating the need for focused education and improved clinical pathways to enhance CV risk management in RMD care.

**WHAT IS ALREADY KNOWN ON THIS TOPIC**

• People with inflammatory rheumatic and musculoskeletal diseases (RMDs) have an increased risk of cardiovascular (CV) disease, driven by both traditional risk factors and disease-related inflammation.
• EULAR has published recommendations to guide healthcare providers (HCPs) in assessing and managing CV risk in individuals with RMDs.
• Despite the availability of these recommendations, the extent to which HCPs are aware of them and the barriers that hinder their implementation remain unclear.

**WHAT THIS STUDY ADDS**

• This international survey shows that although a majority of HCPs report awareness of the EULAR CV recommendations, actual knowledge of key content remains modest.
• Awareness was significantly higher among rheumatologists and more experienced clinicians, but knowledge of the recommendations did not differ substantially by profession or years of experience.
• Lack of dedicated clinical time, insufficient knowledge, and absence of local implementation protocols were the most frequently reported barriers to applying the recommendations in practice.

**HOW THIS STUDY MIGHT AFFECT RESEARCH, PRACTICE OR POLICY**

• The gap between awareness and practical knowledge of CV recommendations highlights the need for targeted educational strategies – such as user-friendly tools, case-based learning, and continuous professional development.
• Institutional-level initiatives—such as integrated CV screening workflows, multidisciplinary collaboration, and electronic health record prompts—may improve implementation of guideline based CV risk management in RMD care.
• Findings may inform future EULAR implementation strategies, guideline dissemination approaches, and development of certification or training programs to strengthen CV risk management in rheumatology.

Alt-text: Unlabelled box dummy alt text

## INTRODUCTION

Inflammatory rheumatic and musculoskeletal diseases (RMDs) are characterised by chronic systemic inflammation and are associated with a substantially increased risk of cardiovascular (CV) disease, which remains a leading cause of morbidity and mortality in this population [[1], [2], [3], [4], [5], [6], [7], [8], [9], [10]].

The underlying mechanisms linking inflammation to CV pathology are multifaceted, encompassing traditional risk factors such as hypertension and dyslipidaemia, as well as disease-specific factors including systemic inflammation, immune dysregulation, and the adverse effects of certain pharmacological therapies, particularly corticosteroids [[11], [12], [13]].

To address this increased CV risk, the European Alliance of Associations for Rheumatology (EULAR) developed recommendations to guide healthcare providers (HCPs) in managing CV risk in people with RMDs [14,15]. These recommendations emphasise the importance of regular CV risk assessment, lifestyle modifications, and the optimisation of pharmacological treatments to mitigate the increased CV risk. Specifically, EULAR places responsibility on the rheumatologist to lead this multidisciplinary effort that also involves cardiologists, primary care physicians, and HCPs in rheumatology, to ensure that patients receive holistic care that addresses both their rheumatologic and CV health needs [15].

Although data suggest some improvement in CV outcomes among patients with RMDs mainly due to newer therapeutic strategies targeting inflammation, CV disease remains a leading cause of death in this population. Despite the publication of EULAR recommendations, the extent of HCPs’ awareness and the presence of potential barriers to implementation of these recommendations into clinical practice remain largely unknown.

Limited awareness, competing clinical priorities, and structural barriers may contribute to suboptimal CV risk assessment and management [[16], [17], [18], [19], [20]].

Given this context, the objective of this study was to assess healthcare professionals’ awareness and understanding of the EULAR CV recommendations, as well as perceived barriers to their implementation in clinical practice among those treating patients with RMDs.

## METHODS

### Study design and setting

We conducted a cross-sectional survey to assess the awareness and implementation of the EULAR recommendations among HCPs involved in the care of patients with RMDs worldwide, though primarily in Europe. The survey was developed and administered using SurveyXact, an online platform that facilitates data collection. The study was endorsed by the EULAR Cardiovascular Involvement in Inflammatory Arthritis (CVIIA) study group, highlighting its relevance and importance within the rheumatology community. The survey was distributed between April and December 2024 using snowball sampling via direct email invitations to professional networks and social media platforms. This approach was chosen to maximise reach and participation across different healthcare professional groups and geographical regions. Participants were encouraged to share the survey link with colleagues. This approach allowed for an assessment of HCPs’ knowledge and perceived barriers to implementing these critical guidelines.

### Survey development

The survey was developed to assess the awareness of the EULAR 2015/2016 and 2022 recommendations [14,15]. It comprised 3 main sections: the first collected demographic and professional information, including sex, age, year of graduation, professional position, years of experience in rheumatology, and country of practice (6 questions); the second and third sections assessed knowledge of the EULAR recommendations, covering awareness, use of CV risk scores, frequency of risk assessment, treatment practices, disease-specific CV risks, and exploration of potential barriers to implementation (16 questions for the 2022 recommendations and 13 questions for the 2015/2016 update).

The survey included predefined multiple-choice items (single- and multiple-response) where applicable, yes/no questions, slider bars for scaled responses (0-100), and free-text fields. For items where more than 1 response was possible, this was explicitly described. Response options for CV-related questions were in a random order to minimise order bias.

The questionnaire was designed to be anonymous, and no identifiable personal data were collected. Participation was voluntary. The survey was available exclusively in English. Questionnaire development was supervised by the author group, all of whom have expertise in rheumatology, CV risk management, and survey studies. Iterative feedback from all authors was used to refine wording, structure, and content to ensure clarity and alignment with the study objectives. Although the questionnaire was not formally piloted in a separate sample, the internal review process was intended to support face and construct validity. The estimated completion time was 10 to 15 minutes. The full questionnaire is provided as Supplementary Material S1.

### Participants and recruitment

The target population for the survey included rheumatology trainees, specialty registrars, rheumatologists, and other HCPs involved in the management of people with RMDs. Recruitment was conducted via direct email invitations to professional networks and social media platforms to maximise participation and ensure a representative sample. A total of 751 invitations were sent out through these combined modalities. To maintain engagement and focus on data collection**,** regular reminders and updates were posted on social media and sent via email throughout the survey period.

### Data collection procedures

Data collection was conducted entirely online via the SurveyXact platform. Participation was voluntary and anonymous, and participants were informed about the purpose of the study at survey entry. Implied informed consent was obtained through completion of the questionnaire.

### Statistical analysis

The statistical software programme Stata version 18.0 (StataCorp LLC, College Station, TX) was used to process and analyse the survey data. A total of 363 responses were received, of which 226 were complete (all questions answered) and were included in the final analysis. The remaining 137 partial responses were excluded; among these, 57% discontinued the survey at or before question 12 of 35, and more than 80% discontinued before question 17. The first 6 questions were purely demographic. Descriptive statistics summarised respondents’ demographic and professional characteristics, including age, sex, year of graduation, professional position, years of experience, and geographic region, with counts and percentages reported. Age, graduation year, and experience were categorised into ranges, and geographic location was grouped into 6 continental regions. Baseline characteristics were summarised in Table 1.

**Table 1:** Baseline characteristics

Characteristics	Total N = 226 (100%)
Age (y)	
<30	23 (10%)
31-45	101 (45%)
46-60	77 (34%)
>60	25 (11%)
Sex	
Male	76 (34%)
Female	148 (66%)
Prefer not to disclaim	1 (0, 4%)
Other	1 (0, 4%)
Graduation year	
Before 1980	4 (2%)
1981-2000	54 (24%)
2000-Present	149 (66%)
Not yet specialised	19 (8)
Position	
Rheumatologist (hospital)	122 (54%)
Rheumatologist (private practice)	10 (4%)
General practitioner/primary care physician	3 (1%)
Resident/specialty registrar	26 (12%)
Junior doctor	5 (2%)
Nurse with experience in rheumatology	37 (16%)
Physiotherapist	6 (3%)
Occupational therapist	3 (1%)
Other	14 (6%)
Years of experience	
<5	54 (24%)
5-14	82 (36%)
15-25	23 (10%)
>25	67 (30%)
Continent	
North America	2 (1%)
South America	2 (1%)
Europe	189 (84%)
Africa	1 (0, 4%)
Asia	32 (14%)

Data are presented as N (%).

Awareness of the 2015/2016 and 2022 EULAR CV recommendations was assessed using survey items Q2_1 and Q3_1 (Supplementary Material S1). Respondents were classified as aware of both recommendation sets, aware of 1 set, or aware of neither. Knowledge was assessed using multiple-choice questions (Q2_2–Q2_14 and Q3_2–Q3_13), with each item scored as correct or incorrect. Total knowledge scores and percentages of correct scores were calculated for each respondent. Barriers to implementation (Q2_16 and Q3_13) were summarised descriptively, and combined variables were created to indicate whether a barrier was selected in either survey section.

To examine associations between professional characteristics and knowledge or awareness, respondents were grouped by specialty (rheumatologists vs nonrheumatologists) and years of experience (<5, 5-15, >15 y). Differences in knowledge scores were assessed using independent *t*-tests for 2-group comparisons and one-way analysis of variance with Bonferroni post hoc tests for 3-group comparisons. Associations between categorical variables, such as awareness by specialty or experience, were assessed using chi-square (χ²) tests, with significance set at *P* < .05.

## RESULTS

### Participant demographics

A total of 363 respondents started completing the survey and a total of 226 complete responses were collected and analysed (resulting in a response rate based on started surveys of approximately 62%). The demographic breakdown of respondents is illustrated in Table 1. A total of 189 of the respondents were based in Europe (84%), and 122 were rheumatologists (54%) working primarily in hospital settings. The educational background of the respondents indicated that 149 (66%) graduated between 2000 and 2024, while 82 (36%) reported having 5 to 15 years of clinical experience.

### Awareness and knowledge of CV recommendations

**Figure 1:**
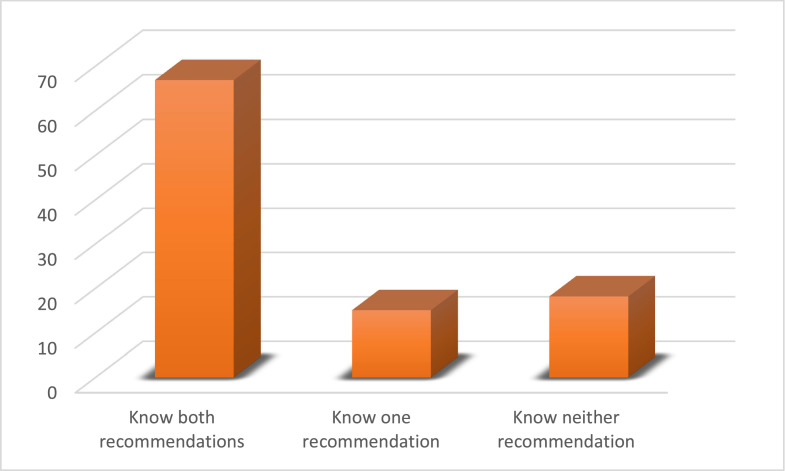
Awareness of the recommendations ‘EULAR recommendations for cardiovascular risk management in rheumatic and musculoskeletal diseases, including systemic lupus erythematosus and antiphospholipid syndrome’ published in 2022 and ‘EULAR recommendations for cardiovascular disease risk management in patients with rheumatoid arthritis and other forms of inflammatory joint disorders: 2015/2016 update’. Two questions were answered by 226 respondents: ‘Are you aware of the updated ‘EULAR recommendations for cardiovascular risk management in rheumatic and musculoskeletal diseases, including systemic lupus erythematosus and antiphospholipid syndrome’ published in 2022?‘ and ‘Are you aware of the ‘EULAR recommendations for cardiovascular disease risk management in patients with rheumatoid arthritis and other forms of inflammatory joint disorders: 2015/2016 update?’ Awareness categories are shown on the x-axis and percentages of respondents (n = 226) on the y-axis. EULAR, European Alliance of Associations for Rheumatology.

**Figure 2:**
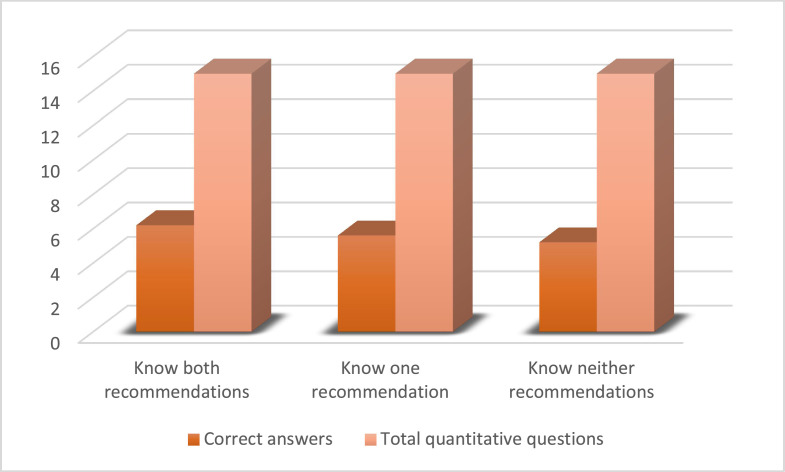
Quantitative questions answered correctly out of 15 based on knowledge of ‘EULAR recommendations for cardiovascular disease risk management in patients with rheumatoid arthritis and other forms of inflammatory joint disorders: 2015/2016 update’ and ‘EULAR recommendations for cardiovascular risk management in rheumatic and musculoskeletal diseases, including systemic lupus erythematosus and antiphospholipid syndrome’ 2022 recommendations. Mean knowledge score (number of correctly answered knowledge questions out of 15) stratified by self-reported awareness of the EULAR cardiovascular risk management recommendations. Dark bars represent mean correct answers, and light bars indicate the maximum possible score (15). EULAR, European Alliance of Associations for Rheumatology.

Overall, awareness of these recommendations varied: 151 respondents (67%) reported familiarity with both sets of recommendations, while 34 (15%) were aware of only 1 set and 41 (18%) indicated no awareness of either (Fig 1). The distribution of total knowledge scores was assessed using the Shapiro–Wilk test. Scores among respondents aware of both EULAR recommendations deviated from normality (*P* < .001). Therefore, a nonparametric test (Mann-Whitney *U*) was used for group comparison. Those familiar with both recommendation sets scored a mean of 6.2 (41%) ± 1.4 compared with 5.2 (35%) ± 1.6 among those unaware of both. The difference was statistically significant (Wilcoxon rank-sum test, z = 4.12, *P* < .001) (Fig 2).

Regarding individual knowledge items, 187 respondents (83%) correctly identified that platelet inhibitors are not recommended for primary CV prevention in individuals with RMDs. Eighteen respondents (8%) correctly indicated the significance of maintaining low disease activity in patients with systemic lupus erythematosus (SLE) to mitigate CV risk, and 216 respondents (96%) acknowledged the necessity of minimising corticosteroid doses to reduce CV harm. Sixteen percent (36 respondents) correctly recognised that CV risk prediction models for rheumatoid arthritis (RA) require application of a 1.5 multiplication factor. None of the respondents (0%) selected the correct option indicating that antihypertensive and lipid-lowering therapies should be applied as in the general population in patients with RA, ankylosing spondylitis, or psoriatic arthritis. For figures presenting these data, see Supplementary Material S2.

To explore whether awareness of the EULAR recommendations was associated with differences in knowledge and practice-related responses, subsequent analyses were stratified according to respondents’ self-reported awareness status. These analyses were restricted to respondents who reported awareness of one or both recommendation sets (n = 151) or no awareness (n = 41); percentages are therefore presented within these subgroups.

When stratified by awareness status, correct identification that platelet inhibitors are not recommended for primary CV prevention was reported by 129 of 151 (85.4%) respondents who reported awareness of both recommendation sets and by 32 of 41 respondents (78.0%) who reported no awareness.

Correct identification of the importance of maintaining low disease activity in patients with SLE was reported by 9 of 151 (6.0%, aware of both) respondents and by 6 of 41 (14.6%, no awareness) respondents.

Acknowledgement of the need to minimise corticosteroid doses to reduce CV harm was reported by 150 of 151 (99.3%, aware of both) respondents and by 39 of 41 (95.1%, no awareness) respondents. Recognition of the need to apply a 1.5 multiplication factor to CV risk prediction models in RA was reported by 18 of 151 (11.9%, aware of both) respondents and by 11 of 41 (26.8%, no awareness) respondents.

### Barriers to implementation

In contrast to the knowledge-based items, the following questions addressed respondents’ perceptions of real-life clinical practice and the contextual factors influencing implementation of the EULAR recommendations.

Questions on key barriers to the implementation of the 2022 recommendations allowed participants to select one or more options from a predefined list of barriers and provide additional free-text responses under ‘Other’. The most frequently cited barrier was ‘a lack of dedicated time in clinical practice’, reported by 141 respondents (62%). In addition, 84 respondents (37%) indicated ‘insufficient knowledge regarding the specific content of the recommendations’ as a significant obstacle, and 31 respondents (14%) noted ‘the absence of these recommendations in local clinical guidelines’ as a barrier to effective implementation; the 2015/2016 recommendations yielded very similar results, see Table 2.

**Table 2:** Reasons for not using EULAR recommendations for cardiovascular risk management among healthcare professionals caring for people with RMDs

Answer	2015/2016 recommendations N (%)^a^	2022 recommendations N (%)^a^
My own lack of knowledge of the specific content of the guideline	77 (34)	84 (37)
Lack of dedicated time clinic	147 (65)	141 (62)
Lack of equipment to measure blood pressure in clinic	15 (7)	12 (5)
Not a part of the clinical guideline in my unit	33 (15)	31 (14)
Unable to perform cholesterol blood tests in clinic	13 (8)	13 (8)
Patients not interested	32 (15)	27 (12)
Other	39 (17)	40 (18)

EULAR, European Alliance of Associations for Rheumatology; RMD, rheumatic and musculoskeletal disease.

a More than 1 reason could be chosen therefore each percentage is relative to 100% of the respondents.

Half of the respondents (50%) indicated that rheumatologists are primarily responsible for CV risk management in patients with inflammatory joint disease, reflecting perceived clinical responsibility in routine practice. When stratified by awareness status, the perception that rheumatologists are primarily responsible for CV risk management was reported by 89 of 151 (58.9%) respondents who reported awareness of the recommendations and by 12 of 41 (29.3%) respondents who reported no awareness. For a figure presenting these data, see Supplementary Material S2.

### Subgroup comparisons

Of 226 respondents, 132 (58%) were rheumatologists, of which 122 (54%) worked in hospital settings and 10 (4%) in private practice. The remaining 94 respondents (42%) represented other professional groups: 26 (12%) residents or specialty registrars, 37 (16%) nurses with experience in rheumatology, 6 (3%) physiotherapists, 3 (1%) occupational therapists, 3 (1%) general practitioners or primary care physicians, 5 (2%) junior doctors, and 14 (6%) classified as other HCPs.

Awareness of the EULAR recommendations differed significantly between professional groups (*P* < .001). Among rheumatologists, 80% (105/132) reported awareness of both recommendation sets, 11% (15/132) reported awareness of 1 set, and 9% (12/132) reported no awareness. Among allied HCPs or nonspecialist doctors, corresponding proportions were 49% (46/94), 20% (19/94), and 31% (29/94), respectively.

Knowledge scores were slightly higher among rheumatologists (mean 6.1 [41%]) compared with allied HCPs or nonspecialists (mean 5.7 [38%]), but this difference was not statistically significant (*P* = .063). When examined by awareness category, mean knowledge scores among rheumatologists were 6.3 among respondents aware of both recommendation sets, 5.5 among those aware of 1 set, and 5.5 among those reporting no awareness. Corresponding mean scores among allied HCPs or nonspecialists were 6.2, 5.6, and 5.1, respectively.

Respondents had <5 years (n = 44), 5 to 14 years (n = 84), 15 to 25 years (n = 56), or >25 years (n = 42) of professional experience. Awareness of both EULAR recommendation sets increased with experience (*P* = .01): 43% of respondents with <5 years of experience (19 aware of both, 9 of one, 16 of none), 70% among those with 5 to 14 years (59 of both, 13 of one, and 12 of none), 71% among those with 15 to 25 years (40 of both, 8 of one, and 8 of none), and 79% among those with >25 years of experience (33 of both, 4 of one, and 5 of none).

However, correct answers to the quantitative survey questions did not differ significantly by experience group: respondents with <5 years of experience answered a mean of 5.77 of 15 questions correctly (38.5%), those with 5 to 15 years answered 5.88 (39.2%), those with 15 to 25 years answered 6.29 (41.9%), and those with >25 years answered 5.83 (38.9%) (*P* = .662). When stratified by awareness category, mean knowledge scores among respondents with <5 years of experience were 5.9 (aware of both), 5.7 (aware of one), and 5.7 (no awareness). Among respondents with 5 to 14 years of experience, corresponding mean scores were 6.4, 5.2, and 4.3; among those with 15 to 25 years, 6.3, 6.9, and 5.5; and among those with >25 years of experience, 6.1, 4.0, and 5.4.

Thus, higher experience level was associated with higher awareness, but not with significantly higher knowledge scores.

## DISCUSSION

Our survey-based study explored CV risk awareness among HCPs in relation to the 2015/2016 and 2022 EULAR [14,15]. This is the first study to address the awareness of these specific recommendations.

In general, awareness of the EULAR CV recommendations was high among respondents. Specifically, 173 of 226 respondents (76.5%) were aware of the 2015/2016 recommendations and 163 of 226 (72.1%) were aware of the 2022 recommendations. Overall, 151 of 226 respondents (67%) were aware of both documents. The difference in awareness of the 2 recommendations was not statistically significant

(McNemar test, χ² = 2.94, *P* = .086), indicating similar recognition despite the differences in publication year.

However, awareness in this context represents self-reported familiarity and does not necessarily reflect a detailed understanding of the recommendations or their correct clinical application.

Consistent with this distinction, despite relatively high awareness, objective knowledge levels were modest.

Respondents who were aware of both recommendations scored a mean of 6.2 ± 1.4 of 15 questions (41%), compared with 5.2 ± 1.6 (35%) among those unaware of both. Several explanations may account for this pattern. Greater professional experience may increase recognition of guideline documents, while detailed recall of specific recommendations may diminish without regular reinforcement. Conversely, more recently trained clinicians may demonstrate comparable knowledge due to integration of guideline-based CV risk management as part of contemporary training.

Stratification by self-reported awareness status further clarified the relationship between guideline familiarity, knowledge, and practice-related perceptions. Several broadly accepted principles, such as avoidance of platelet inhibitors for primary CV prevention and minimisation of corticosteroid exposure, were correctly identified by a substantial proportion of respondents who reported no awareness of the EULAR recommendations, suggesting that some aspects of CV risk management may reflect general clinical knowledge rather than direct recommendation familiarity. In contrast, more specific recommendation elements requiring detailed recall, such as application of the 1.5 multiplication factor for CV risk prediction in RA, were poorly recognised regardless of awareness status. Awareness was, however, associated with differences in perceived responsibility for CV risk management, indicating that guideline familiarity may influence conceptualisation of clinical roles even when detailed knowledge remains limited.

Several individual knowledge items revealed particularly low rates of correct responses, notably those concerning the application of the 1.5 multiplication factor for CV risk prediction in RA and the recommendation to manage antihypertensive and lipid-lowering therapies analogously to the general population. These findings warrant careful interpretation. Although they can reflect genuine gaps in detailed knowledge of specific recommendations, they may also highlight a disconnect between guideline statements and everyday clinical practice. In particular, pharmacological management of CV risk factors is often perceived as the responsibility of primary care physicians or cardiologists, which may influence how rheumatology-focused clinicians interpret and respond to such questions, despite the recommendations stating that CV risk assessment is the responsibility of the rheumatologist.

Subgroup analyses further show the distinction between awareness and applied knowledge. Rheumatologists and more experienced clinicians were more likely to report awareness of the EULAR recommendations, whereas differences in objective knowledge scores between professional groups were small and not statistically significant. Importantly, increasing professional experience was associated with greater awareness but not with higher knowledge scores, even when examined across awareness categories. These findings suggest that professional seniority and specialty may facilitate recognition of guideline documents, but do not necessarily ensure retention or application of specific recommendation content.

Implementation was hindered by barriers such as lack of dedicated time (141 respondents, 62%), insufficient knowledge (84 respondents, 37%), and absence of local protocols (31 respondents, 14%). Importantly, our results demonstrate that awareness does not directly translate into competency or knowledge, highlighting the need for targeted and practical educational materials as part of an implementation framework. This gap may reflect the absence of specific strategies in the current recommendation documents, although future updates under the EULAR umbrella may address this limitation [21].

Our findings have important implications for clinical practice. Given the increased risk of premature CV burden among patients with RMDs, it is crucial for HCPs to implement EULAR recommendations, supported by institutional-level strategies. Barriers such as time constraints, lack of resources, and absence of local protocols indicate a pressing need for system-level adjustments. Enhancing CV risk management as an integrated part of clinical rheumatology practice therefore requires multilevel strategies, including individual HCP education and institutional support through multidisciplinary teams, optimised workflows, and allocation of resources. Individual-level strategies include user-friendly resources, such as quick reference guides, decision support tools, and case-based learning modules. Continuous professional development through workshops and training can further support HCPs in staying updated on the latest guidelines and best practices.

Institutional-level strategies include multidisciplinary approaches—such as CV screening clinics led by nurses or nurse practitioners—and regular meetings to facilitate knowledge sharing and adherence to EULAR recommendations [14,15]. Technology can also aid implementation, for example, through electronic health record (EHR) prompts and mobile apps that provide recommendation access. Educational initiatives and certification programmes can support individual knowledge and competence, but their impact depends on integration with institutional strategies. Combined, these efforts can inform broader educational frameworks beyond rheumatology.

Our findings provide a foundation for designing strategies to improve EULAR recommendation implementation. Targeted educational initiatives should address specific challenges identified in our study, including case-based learning modules that enhance the practical application of recommendations. Educational efforts may also extend to patients and other specialties. Institutions can implement multidisciplinary approaches, such as nurse-led CV screening clinics and regular team meetings, to support holistic care and knowledge sharing [22,23]. Technology, including EHR prompts and mobile guideline apps, can further facilitate guideline uptake. Finally, findings may inform future European Rheumatology certification programmes, where knowledge of recommendations and guidelines is systematically assessed.

### Strengths and weaknesses

The survey presents notable strengths in design and implementation. First, the multicentric recruitment strategy enabled inclusion of a diverse sample of HCPs across Europe, which allowed for capture variation in awareness and knowledge regarding EULAR CV risk recommendations for RMDs. The use of a validated online platform and endorsement by a recognised EULAR study group contribute to credibility and potentially facilitated response rates. The nature of the questionnaire, with both quantitative and qualitative items on barriers to implementation, allowed for a nuanced understanding of factors influencing clinical practice and guideline adherence. Despite these methodological strengths, our study has some weaknesses. The reliance on self-reported data exposes the findings to recall and social desirability bias, with the risk that respondents may overestimate either awareness or adherence to recommendations. Importantly, our results suggest that high reported awareness does not necessarily translate into adequate knowledge or competency, highlighting the need for targeted implementation actions to ensure that recommendation awareness is effectively translated into real-world clinical practice. The survey yielded a response rate of 62% (calculated as the proportion of participants who completed the survey relative to those who started it), which raises the possibility of selection bias, whereby HCPs more engaged or interested in CV risk management may have been disproportionately represented. However, examination of response patterns among incomplete questionnaires showed that most partial responses terminated early in the survey. Although this pattern may indicate that early discontinuation was related to time constraints or survey burden rather than selective dropout after engagement with the knowledge questions, selection bias cannot be fully excluded. This may inflate perceived baseline awareness relative to the wider clinical population. Further, variable participation from different professional groups, such as rheumatologists versus other HCPs, may introduce confounding, given documented differences in recommendation familiarity and scope of clinical practice. In addition, cardiologists, internists, and general practitioners were underrepresented, despite their central role in CV risk management. As a result, the present findings primarily reflect perceptions from a rheumatology-centred perspective and may not fully capture the complexity of multidisciplinary CV care pathways. The cross-sectional nature of the study precludes assessment of changes over time or determination of causality between awareness and implementation practices. Interpretation of subgroup comparisons is limited by sample sizes in less-represented categories. The absence of objective measures of adherence to the recommendations, such as patient notes review or audit of clinical practice, constrains inference about true practice impact. Also, geographic representation was also skewed, as the majority of responses originated from Europe, thereby limiting generalisability to global practice. In addition, the knowledge questions, while carefully constructed, were not externally validated, which may have introduced measurement bias. These methodological caveats highlight the importance of contextualising results within the constraints of survey-based research. They emphasise the need for future studies to leverage a broader sampling frame to substantiate and extend the present findings and, where possible, include objective measures to assess adherence to the recommendations.

The results highlight significant awareness of EULAR recommendations among HCPs regarding CV risk management; however, they also highlight the challenges in translating this awareness into clinical practice. The identified barriers necessitate targeted educational initiatives, multidisciplinary approaches, and the integration of technology to enhance the implementation of the recommendations and improve CV risk management in patients with rheumatic diseases, ultimately leading to better health outcomes in this vulnerable population. These findings may pave the way for further understanding how recommendations are implemented and for the development of effective, multilevel strategies that bridge the knowledge-to-action gap in clinical practice.

## Funding

This research did not receive any specific grant from funding agencies in the public, commercial, or not-for-profit sectors.

## CRediT authorship contribution statement

**Dina Husum:** Writing – review & editing, Writing – original draft, Visualization, Software, Methodology, Investigation, Formal analysis, Data curation. **Julie Katrine Karstensen:** Writing – review & editing. **Massimo Radin:** Writing – review & editing, Supervision. **Savino Sciascia:** Writing – review & editing. **Alessia Alunno:** Writing – review & editing. **Fabiola Atzeni:** Writing – review & editing. **Javier Rodríguez-Carrio:** Writing – review & editing. **Michael T. Nurmohamed:** Writing – review & editing. **Sander I. van Leuven:** Writing – review & editing. **Zoltán Szekanecz:** Writing – review & editing. **Jette Primdahl:** Writing – review & editing. **Karen Schreiber:** Writing – review & editing, Writing – original draft, Supervision, Methodology, Investigation, Conceptualization.

## Competing interests

All authors declare they have no competing interests.

## Appendix Supplementary materials

Image, application 1

Image, application 2

## Footnotes

Supplementary material associated with this article can be found in the online version at doi:10.1016/j.ero.2026.02.015.
