# Supplementary material for: Healthcare providers’ awareness and knowledge of recommendations on cardiovascular risk management in people with rheumatic and musculoskeletal diseases: a survey study
Source: EULAR Rheumatol Open. 2026 Mar 13;2(1):362–9. doi: 10.1016/j.ero.2026.02.015 (PMC13292127; doi:10.1016/j.ero.2026.02.015)
Supplement: Supplementary file 1 [file mmc1.docx]

# Supplementary Material S1

## Global awareness of recommendations on cardiovascular risk management in people with rheumatic and musculoskeletal diseases among healthcare providers - a survey study

# The Survey

## Introduction

**Global awareness of EULAR (The European Alliance of Associations for Rheumatology) recommendations for cardiovascular risk management in patients with inflammatory rheumatic diseases – a survey study**

This questionnaire is designed to explore your knowledge and awareness of the ‘**EULAR recommendations for cardiovascular risk management in rheumatic and musculoskeletal diseases, including systemic lupus erythematosus and antiphospholipid syndrome**’ (section 2) and ‘**EULAR recommendations for cardiovascular disease risk management in patients with rheumatoid arthritis and other forms of inflammatory joint disorders: 2015/2016 update**’ (section 3) article recommondations.

It is anonymous and will take you 10 min to complete. Thank you for your time!

**This survey is endorsed by the EULAR Cardiovascular involvement in Inflammatory arthritis (CVIIA) study group.*

## Section 1: Demographic Information

1.1. Gender

- Male
- Female
- Prefer not to disclaim
- Other

1.2. Age

- Slidebar from 25 til 100

1.3. 1.3. When did you graduate your specialty/graduate your education (if you are not yet done with your specialty/education, please set timeline at when you are expected to)

- Timeline, numerical choice (1960-2030)

1.4. Position

- Rheumatologist (Hospital)
- Rheumatologist (Private practice)
- General practitioner/Primary Care Physician
- Resident/Specialty Registrar
- Junior Doctor
- Nurse with experience in rheumatoid patients
- Physiotherapist
- Occupational therapist
- Other (free text)

1.5. Years of experience in Rheumatology

- Slidebar from 1 to 30

1.6. Country of Practice:

- Alphabetical order all countries

## Section 2: Overarching principles and recommendations

The following questions refer to the ‘**EULAR recommendations for cardiovascular risk management in rheumatic and musculoskeletal diseases, including systemic lupus erythematosus and antiphospholipid syndrome**’ published in 2022 (G. Drosos et al. ARD 2022)

- 1. Are you aware of the updated ‘EULAR recommendations for cardiovascular risk management in rheumatic and musculoskeletal diseases, including systemic lupus erythematosus and antiphospholipid syndrome’ published in 2022?
- Yes
- No
  1. Who is responsible for the cardiovascular risk (CVR) assessment and management?
- Primary care providers
- Internists
- Cardiologists
- Other healthcare providers
- **A collaboration between 1-4**
  1. The guideline states: *“In patients with gout, vasculitis, SSc, myositis, MCTD and SS, we recommend thorough assessment of traditional CVR factors. The use of cardiovascular prediction tools as for the general population is recommended.”* These include the Framingham Risk Score (FRS), QRISK3 or Systematic Coronary Risk Evaluation (SCORE).

*Systemic Sclerosis (SSc), mixed connective tissue disease (MCTD), Sjögren's Syndrome (SS), Cardiovascular Risk (CVR)

Which score do you use in your practice?

- Framingham Risk Score (FRS)
- QRISK3
- Systematic Coronary Risk Evaluation (SCORE)
- Other (specify)
  1. With your current experience, how often should RMD patients be screened for the presence of cardiovascular risk factors?
- Every month
- Every 6 months
- 6 months after diagnosis and then never again
- **6 months after diagnosis and then regularly depending on the patients risk level**
  1. In your opinion, how important is patient education, counselling on cardiovascular risk (CVR), treatment adherence and lifestyle modifications, such as healthy diet and regular physical activity important in the management of CVR in an RMD patient?

Slidebar 🡪 not important 0 – most important 100

​​​​​​2.6 For which rheumatic diseases might the Framingham score be underestimating the cardiovascular risk?

*NB: Information from the EUVAS (European Vasculitis Society) model may supplement modifiable Framingham risk factors and is recommended by the article to take into account*

- Gout
- Sjögren's Syndrome/Disease
- Systemic Sclerosis
- **ANCA-associated vasculitis**
- All of the above

​​​​​2.7 In patients with gout which medications should be avoided?

- Digoxin
- **Diuretics**
- Calcium-antagonists
- β-blockers
- I do not have a license to prescribe medicine

​​​​​2.8 In patients with Systemic Sclerosis (SSc) which medications should be avoided?

- **β-blockers**
- Diuretics
- Calcium-antagonists
- Digoxin
- I do not have a license to prescribe medicine.

​​​​​​2.9 In patients with gout, vasculitis, Systemic Sclerosis (SSc), myositis, mixed connective tissue disease (MCTD) and/or Sjögren’s Syndrome (SS) is the use of platelet inhibitors recommended for primary CVD prevention?

- **No, it is not recommended. Treatment with platelet inhibitors should follow recommendations used in the general population**
- Yes, it is recommended, and should be given upon diagnosis in all patients above

​​​​​2.10 In your personal opinion, how much do you think disease remission induction and remission maintenance in patients with ANCA-associated vasculitis reduce the patients cardiovascular risk?

Slidebar 🡪 0 (not at all) to 100 (a lot)

​​​​​​2.11 In patients with SLE, lower levels of blood pressure are associated with lower rates of cardiovascular events. What is the target blood pressure?

- The target blood pressure should be <140/90 mm Hg
- **The target blood pressure should be <130/80 mm Hg**

​​​​​2.12 In which patients is prophylactic low-dose aspirin recommended?

- Asymptomatic persistent antiphospholipid antibody (aPL) carriers (not fulfilling any vascular or obstetric antiphospholipid Syndrome (APS) classification criteria) with a high-risk aPL profile with or without traditional risk factors
- In patients with Systemic Lupus Erythematosus (SLE) with no history of thrombosis or pregnancy complications with high-risk aPL profile
- **Both answers above include a consideration on patients with SLE and no history of thrombosis or pregnancy complications with low-risk aPL profile**

2.13 Assessing the cardiovascular risk (CVR), does diseases activity matter in patients with Systemic Lupus Erythematosus (SLE)?

- No, the disease activity does not correlate with CVR
- **Yes, low disease activity should be maintained to reduce CVR**
- Yes, medium disease activity should be maintained to reduce CVR

2.14 ​​​​​Does the dose of corticosteroids in SLE patients have any correlation to cardiovascular harm?

- No, there is no correlation
- **Yes, they should be treatment with the lowest possible corticosteroid dose to minimize any potential cardiovascular harm**

2.15 Please estimate the percentage of patients in whom you assess cardiovascular risk as per EULAR 2022 guidelines in patients with rheumatic and musculoskeletal diseases, including systemic lupus erythematosus and antiphospholipid syndrome?

- Slidebar from 1 to 100 %

2.16 What are your reasons for not using the guidelines? (tick as many as apply, answers are anonymous)

- My own lack of knowledge of the specific content of the guidelines
- Lack of dedicated time in clinic
- Lack of equipment to perform blood pressure in clinic
- Unable to perform cholesterol blood tests in clinic
- Patients not interested
- Other reasons

## Section 3: Overarching principles and recommendations

The following questions refer to the ‘**EULAR recommendations for cardiovascular disease risk management in patients with rheumatoid arthritis and other forms of inflammatory joint disorders: 2015/2016 update**’ published in 2016 (R. Agca et al. ARD 2017)

​​​​​3.1 Are you aware of the ‘EULAR recommendations for cardiovascular disease risk management in patients with rheumatoid arthritis and other forms of inflammatory joint disorders: 2015/2016 update’?

- Yes
- No

​​​​​​3.2 Are you aware of the increased risk of cardiovascular disease (CVD) in patients with rheumatoid arthritis (RA) compared to the general population?

- Yes, but I don't consider it a significant factor
- Yes, and I recognize it as an important consideration in patient care
- No, I believe their risk is similar to the general population
- Yes, but I am unsure of its impact on patient management

​​​​​3.3 Who do you believe is *primarily* responsible for managing cardiovascular disease (CVD) risk in patients with inflammatory joint disorders (IJD)?

- Primary care physicians
- Cardiologists
- **Rheumatologists**
- Endocrinologists

​​​​​3.4 Do you follow treatment-specific recommendations from EULAR and other relevant societies regarding the use of NSAIDs and corticosteroids in patients with rheumatoid arthritis (RA) and inflammatory joint disorders?

- No, I rely solely on my clinical judgment
- Yes, I adhere strictly to the provided guidelines
- No, I am not aware of any specific recommendations
- Yes, but I often deviate from the recommendations

​​​​​3.5 How often should you perform cardiovascular disease (CVD) risk assessment for patients with rheumatoid arthritis (RA), ankylosing spondylitis (AS), or psoriatic arthritis (PsA)?

- Never
- Every 10 years (and reconsidered following major changes in antirheumatic therapy)
- **Every 5 years (and reconsidered following major changes in antirheumatic therapy)**
- Every time they visit (and reconsidered following major changes in antirheumatic therapy)

​​​​​3.6 How do you assess cardiovascular disease (CVD) risk estimation for patients with rheumatoid arthritis (RA), ankylosing spondylitis (AS), or psoriatic arthritis (PsA)?

- I never assess the CVD risk
- I assess using my clinical knowledge
- I assess according to national guidelines (or the SCORE CVD risk prediction model if no national guideline is available)

3.7 ​​​​​Do you emphasize lifestyle recommendations such as healthy diet, regular exercise, and smoking cessation when discussing cardiovascular disease (CVD) risk management with your patients?

- Yes, whenever possible
- No, I believe medication alone is sufficient
- No, I do not believe lifestyle changes affect CVD risk

​​​​​3.8 When should lipids ideally be measured for cardiovascular disease (CVD) risk assessment in patients with rheumatoid arthritis (RA), ankylosing spondylitis (AS), and psoriatic arthritis (PsA), according to the EULAR recommendations?

- Lipids should be measured during disease activity regardless of stability or remission
- Lipids should be measured only during acute disease flares
- **Lipids should ideally be measured when disease activity is stable or in remission**

​​​​​​3.9 How should cardiovascular disease (CVD) risk prediction models be adjusted for patients with rheumatoid arthritis (RA), according to the EULAR recommendations?

- No adjustment is necessary; CVD risk prediction models are already accurate for patients with RA
- **CVD risk prediction models should be adapted for patients with RA by a 1.5 multiplication factor, if this is not already included in the model**
- CVD risk prediction models should be adjusted by a multiplication factor of 3.2 for patients with RA

​​​​​3.10 How should antihypertensives and statins be managed in patients with rheumatoid arthritis (RA), ankylosing spondylitis (AS), or psoriatic arthritis (PsA), according to the EULAR recommendations?

- **Antihypertensives and statins may be used as in the general population**
- You cannot use these treatments in patients with RA, PsA or AS
- Antihypertensives and statins should be used differently for patients with RA, AS or PsA compared to the general population

3.11 ​​​​​Are you cautious in prescribing NSAIDs in patients with rheumatoid arthritis (RA) and psoriatic arthritis (PsA), especially for those with documented cardiovascular disease (CVD) or in the presence of CVD risk factors?

- No, I prescribe NSAIDs routinely regardless of CVD risk factors
- Yes, I prescribe NSAIDs with caution in both RA and PsA patients with documented CVD or CVD risk factors
- Yes, but only for my RA patients not my PsA patients

3.12 Please estimate the percentage of patients in whom you assess cardiovascular risk as per EULAR 2015/16 recommendations for cardiovascular risk management in patients with rheumatoid arthritis and other forms of inflammatory joint disorders?

- Slidebar from 0 to 100

3.13 What are your reasons for not using the guidelines? (tick as many as apply, answers are anonymous)

- My own lack of knowledge of the specific content of the guidelines
- Lack of dedicated time in clinic
- Lack of equipment to perform blood pressure in clinic
- Unable to perform cholesterol blood tests in clinic
- Patients not interested
- Other reasons

## Ending text

Thank you for your valuable input.
