# Supplementary material for: Healthcare providers’ awareness and knowledge of recommendations on cardiovascular risk management in people with rheumatic and musculoskeletal diseases: a survey study
Source: EULAR Rheumatol Open. 2026 Mar 13;2(1):362–9. doi: 10.1016/j.ero.2026.02.015 (PMC13292127; doi:10.1016/j.ero.2026.02.015)
Supplement: Supplementary file 2 [file mmc2.docx]

# Supplementary Material S2

### Global awareness of recommendations on cardiovascular risk management in people with rheumatic and musculoskeletal diseases among healthcare providers - a survey study

| Figure 3Platelet inhibitors as primary CV prevention in patients with RMD |
| --- |
|  |
| A total of 226 respondents answered the question; *‘In patients with gout, vasculitis, Systemic Sclerosis (SSc), myositis, mixed connective tissue disease (MCTD) and/or Sjögren’s Syndrome (SS) is the use of platelet inhibitors recommended for primary CVD prevention? No, it is not recommended. Treatment with platelet inhibitors should follow recommendations used in the general population’* or *‘Yes, it is recommended, and should be given upon diagnosis in all patients above’.* 187 answered *“No, …”* correctly. |

| Figure 4Disease activity matters in patients with systemtic lupus erythematosus |
| --- |
|  |
| A total of 226 respondents answered the question: *‘Assessing the cardiovascular risk (CVR), does diseases activity matter in patients with Systemic Lupus Erythematosus (SLE)?’ ‘No, the disease activity does not correlate with CVR’* or *‘Yes, low disease activity should be maintained to reduce CVR’* or *‘Yes, medium disease activity should be maintained to reduce CVR’.* Correctly answered by 18 respondents as *“Yes, low…”*. |

| Figure 5Dose of corticosteroids correlation to CV harm in patients with systemic lupus erythematosus |
| --- |
|  |
| A total of 226 respondents answered the question: *Does the dose of corticosteroids in SLE patients have any correlation to cardiovascular harm?’ ‘No, there is no correlation’* or ‘*Yes, they should be treatment with the lowest possible corticosteroid dose to minimize any potential cardiovascular harm’.* Correctly answered by 218 respondents as *“Yes, …”*. |

| Figure 6Who is primarily responsible for CV risk management in patients with inflammatory joint disorders |
| --- |
|  |
| A total of 226 respondents answered the question; *‘Who do you believe is primarily responsible for managing cardiovascular disease (CVD) risk in patients with inflammatory joint disorders (IJD)?’ ‘Primary care physicians’* or *‘Cardiologists’* or *‘Rheumatologists’* or *‘Endocrinologists’.* Correctly answered by 112 respondents as ‘*Rheumatologists’*. |

| Figure 7How should CV prediction models be adjusted for patients with rheumatoid arthritis |
| --- |
|  |
| A total of 226 respondents answered the question: *‘How should cardiovascular disease (CVD) risk prediction models be adjusted for patients with rheumatoid arthritis (RA), according to the EULAR recommendations?’ ‘No adjustment is necessary; CVD risk prediction models are already accurate for patients with RA’* or *‘CVD risk prediction models should be adapted for patients with RA by a 1.5 multiplication factor, if this is not already included in the model’* or *‘CVD risk prediction models should be adjusted by a multiplication factor of 3.2 for patients with RA’.* Correctly answered by 36 respondents as ‘*CVD risk…by a 1,5 multiplication factor, …’*. |

| Figure 8Antihypertensives and statins management in patients with rheumatoid arthritis, ankylosing spondylitis and psoriatric arthrtitis |
| --- |
|  |
| A total of 226 respondents answered the question: *‘How should antihypertensives and statins be managed in patients with rheumatoid arthritis (RA), ankylosing spondylitis (AS), or psoriatic arthritis (PsA), according to the EULAR recommendations?’ ‘Antihypertensives and statins may be used as in the general population’* or *‘You cannot use these treatments in patients with RA, PsA or AS’* or *‘Antihypertensives and statins should be used differently for patients with RA, AS or PsA compared to the general population’.* Correctly answered by 0 respondents as ‘*Antihypertensives and statins may be used as in the general population’*. |
